# (2*Z*,3*Z*)-Quinoxaline-2,3(1*H*,4*H*)-dione dioxime

**DOI:** 10.1107/S1600536808021570

**Published:** 2008-07-16

**Authors:** Ali Kakanejadifard, Vahid Amani

**Affiliations:** a Department of Chemistry, Faculty of Science, Lorestan University, Khorramabad, Iran; b Department of Chemistry, Islamic Azad University, Shahr-e-Rey Branch, Tehran, Iran

## Abstract

The asymmetric unit of the title compound, C_8_H_8_N_4_O_2_, contains one half-mol­ecule; a twofold rotation axis bisects the molecule. An intra­molecular N—H⋯O hydrogen bond results in the formation of a five-membered ring, which displays an envelope conformation. In the crystal structure, inter­molecular O—H⋯N hydrogen bonds link the mol­ecules.

## Related literature

For related literature, see: Kakanejadifard, Niknam & Zabardasti (2007 ▶); Kakanejadifard, Saniei *et al.* (2007 ▶); Kakanejadifard & Niknam (2006 ▶); For general background, see: Jones *et al.* (1961 ▶); Schrauzer & Kohnle (1964 ▶); Yari *et al.* (2006 ▶); Hashemi *et al.* (2006 ▶); Ghiasvand *et al.* (2004 ▶, 2005 ▶); Kakanejadifard, Niknam, Ranjbar *et al.* (2007 ▶); Gok & Kantekin (1997 ▶).
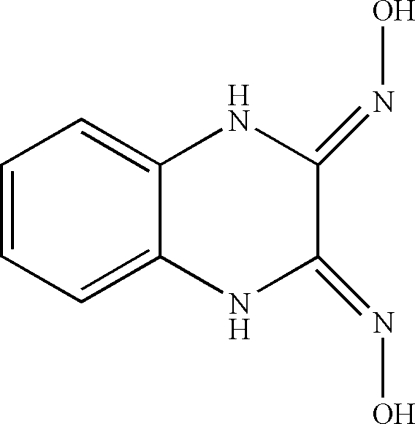


## Experimental

### 

#### Crystal data

C_8_H_8_N_4_O_2_
*M* *_r_* = 192.18
Orthorhombic, 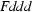

*a* = 9.831 (2) Å
*b* = 13.609 (3) Å
*c* = 24.344 (5) Å
*V* = 3256.9 (11) Å^3^
*Z* = 16
Mo *K*α radiation
μ = 0.12 mm^−1^
*T* = 120 (2) K
0.4 × 0.2 × 0.2 mm

#### Data collection

Bruker SMART 1000 CCD area-detector diffractometer
Absorption correction: none
7925 measured reflections
983 independent reflections
708 reflections with *I* > 2σ(*I*)
*R*_int_ = 0.065

#### Refinement

*R*[*F* ^2^ > 2σ(*F* ^2^)] = 0.060
*wR*(*F* ^2^) = 0.118
*S* = 1.01
983 reflections
67 parameters
1 restraint
H atoms treated by a mixture of independent and constrained refinement
Δρ_max_ = 0.43 e Å^−3^
Δρ_min_ = −0.25 e Å^−3^

### 

Data collection: *SMART* (Bruker, 1998 ▶); cell refinement: *SMART*; data reduction: *SAINT-Plus* (Bruker, 1998 ▶); program(s) used to solve structure: *SHELXTL* (Sheldrick, 2008 ▶); program(s) used to refine structure: *SHELXTL*; molecular graphics: *SHELXTL*; software used to prepare material for publication: *SHELXTL*.

## Supplementary Material

Crystal structure: contains datablocks I, global. DOI: 10.1107/S1600536808021570/hk2493sup1.cif

Structure factors: contains datablocks I. DOI: 10.1107/S1600536808021570/hk2493Isup2.hkl

Additional supplementary materials:  crystallographic information; 3D view; checkCIF report

## Figures and Tables

**Table 1:** Hydrogen-bond geometry (Å, °)

*D*—H⋯*A*	*D*—H	H⋯*A*	*D*⋯*A*	*D*—H⋯*A*
O1—H1⋯N1^i^	0.852 (13)	1.971 (10)	2.763 (2)	154
N2—H2*A*⋯O1	0.86	2.25	2.566 (3)	102

Symmetry code: (i) 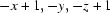.

**Fig. 1.**
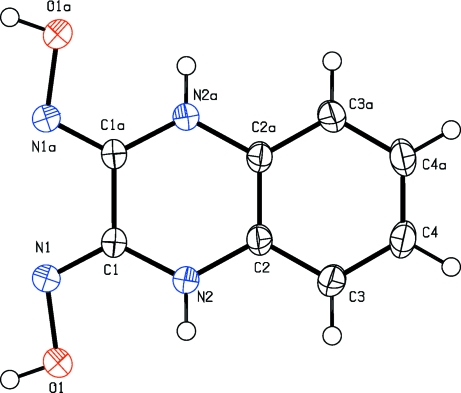
The molecular structure of the title molecule, with the atom-numbering scheme. Displacement ellipsoids are drawn at the 50% probability level [symmetry code: (a) 5/4 - x, 1/4 - y, z].

**Fig. 2.**
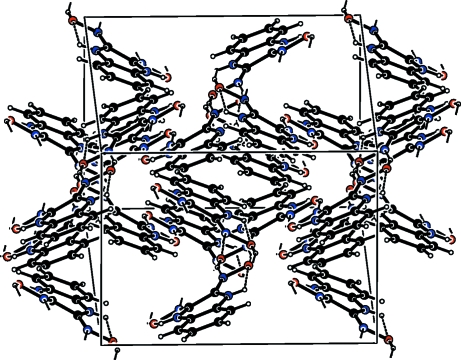
A packing diagram of the title molecule. Hydrogen bonds are shown as dashed lines.

## supplementary crystallographic information

### Comment

Recently, we have reported the syntheses and chemical behaviours of some vic-dioximes. In our investigations, the reactions of amines with dichloro- glyoxime or cyanogendi-N-oxide resulted in various symmetrically substituted diaminoglyoxime derivatives, in which some of them were quite suitable to act, as donor species, towards some transition metal ions (Kakanejadifard, Niknam & Zabardasti, 2007; Kakanejadifard, Saniei *et al.*, 2007; Kakanejadifard & Niknam, 2006). Some oximes are widely used for various purposes in organic, inorganic, bioinorganic, pigment, analytical, dyes and medical chemistry (Jones *et al.*, 1961; Schrauzer & Kohnle, 1964;Yari *et al.*, 2006; Hashemi *et al.*, 2006; Ghiasvand *et al.*, 2004, 2005; Kakanejadifard, Niknam, Ranjbar *et al.*, 2007). vic-Dioximes, containing mildly acidic hydroxyl groups and slightly basic nitrogen atoms, are amphoteric and their transition metal complexes have been widely investigated as analytical reagents (Gok & Kantekin, 1997). We report herein the synthesis and crystal structure of the title compound.

The asymmetric unit of the title compound contains one-half molecule (Fig. 1). The intramolecular N-H···O hydrogen bond (Table 1) results in the formation of a five-membered ring: (O1/N1/N2/C1/H2A), having envelope conformation, with H2A atom displaced by -0.132 Å from the plane of the other ring atoms.

In the crystal structure, intermolecular O-H···N hydrogen bonds (Table 1) link the molecules (Fig. 2), in which they may be effective in the stabilization of the structure.

### Experimental

For the preparation of the title compound, a solution of Na_2_CO_3_ (0.2 g, 1.9 mmol) in MeCN (30 ml) was added to a magnetically stirred solution of dicholoroglyoxime (1.57 g, 10 mmol) in MeCN (20 ml) and a solution of 1,2-phenylendiamine (1.08 g, 10 mmol) at 278 K. After 2 h stirring at room temperature, the mixture was filtered and the brown precipitate was washed with cold MeCN. It was recrystallized from EtOH (70% aq) in one week (yield; 93.0%, m.p. 512 K).

### Refinement

H1 atom (for OH) was located in difference syntheses and refined [O-H = 0.852 (13) Å, U_iso_(H) = 0.039 Å^2^]. The remaining H atoms were positioned geometrically, with N-H = 0.86 Å (for NH) and C-H = 0.93 Å for aromatic H, and constrained to ride on their parent atoms with U_iso_(H) = 1.2U_eq_(C,N).

### Crystal data

**Table:**

C_8_H_8_N_4_O_2_	*F*_000_ = 1600
*M**_r_* = 192.18	*D*_x_ = 1.568 Mg m^−^^3^
Orthorhombic, *F**d**d**d*	Mo *K*α radiation λ = 0.71073 Å
Hall symbol: -F 2uv 2vw	Cell parameters from 744 reflections
*a* = 9.831 (2) Å	θ = 3–30º
*b* = 13.609 (3) Å	µ = 0.12 mm^−^^1^
*c* = 24.344 (5) Å	*T* = 120 (2) K
*V* = 3256.9 (11) Å^3^	Prism, light-brown
*Z* = 16	0.4 × 0.2 × 0.2 mm

### Data collection

**Table:**

Bruker SMART 1000 CCD area-detector diffractometer	708 reflections with *I* > 2σ(*I*)
Radiation source: fine-focus sealed tube	*R*_int_ = 0.065
Monochromator: graphite	θ_max_ = 28.0º
*T* = 120(2) K	θ_min_ = 2.7º
φ and ω scans	*h* = −12→12
Absorption correction: none	*k* = −17→17
7925 measured reflections	*l* = −32→31
983 independent reflections	

### Refinement

**Table:**

Refinement on *F*^2^	Secondary atom site location: difference Fourier map
Least-squares matrix: full	Hydrogen site location: difference Fourier map
*R*[*F*^2^ > 2σ(*F*^2^)] = 0.060	H atoms treated by a mixture of independent and constrained refinement
*wR*(*F*^2^) = 0.118	*w* = 1/[σ^2^(*F*_o_^2^) + (0.005*P*)^2^ + 25*P*] where *P* = (*F*_o_^2^ + 2*F*_c_^2^)/3
*S* = 1.01	(Δ/σ)_max_ < 0.001
983 reflections	Δρ_max_ = 0.43 e Å^−^^3^
67 parameters	Δρ_min_ = −0.25 e Å^−^^3^
1 restraint	Extinction correction: none
Primary atom site location: structure-invariant direct methods	

### Special details

**Table:**

Geometry. All e.s.d.'s (except the e.s.d. in the dihedral angle between two l.s. planes) are estimated using the full covariance matrix. The cell e.s.d.'s are taken into account individually in the estimation of e.s.d.'s in distances, angles and torsion angles; correlations between e.s.d.'s in cell parameters are only used when they are defined by crystal symmetry. An approximate (isotropic) treatment of cell e.s.d.'s is used for estimating e.s.d.'s involving l.s. planes.
Refinement. Refinement of F^2^ against ALL reflections. The weighted R-factor wR and goodness of fit S are based on F^2^, conventional R-factors R are based on F, with F set to zero for negative F^2^. The threshold expression of F^2^ > 2sigma(F^2^) is used only for calculating R-factors(gt) etc. and is not relevant to the choice of reflections for refinement. R-factors based on F^2^ are statistically about twice as large as those based on F, and R- factors based on ALL data will be even larger.

### Fractional atomic coordinates and isotropic or equivalent isotropic displacement parameters (Å^2^)

**Table:**

	*x*	*y*	*z*	*U*_iso_*/*U*_eq_	
O1	0.47468 (17)	−0.04564 (12)	0.43944 (6)	0.0324 (4)	
H1	0.454 (3)	−0.0611 (18)	0.4723 (4)	0.039*	
N1	0.54273 (19)	0.04548 (13)	0.44726 (7)	0.0261 (4)	
N2	0.5691 (2)	0.03307 (14)	0.35156 (7)	0.0308 (5)	
H2A	0.5385	−0.0261	0.3513	0.037*	
C1	0.5867 (2)	0.07818 (15)	0.40084 (8)	0.0242 (4)	
C2	0.5981 (2)	0.07776 (16)	0.30119 (8)	0.0268 (5)	
C3	0.5745 (2)	0.03025 (19)	0.25164 (9)	0.0358 (6)	
H3A	0.5413	−0.0337	0.2514	0.043*	
C4	0.6000 (3)	0.0776 (2)	0.20291 (9)	0.0412 (6)	
H4A	0.5835	0.0457	0.1698	0.049*	

### Atomic displacement parameters (Å^2^)

**Table:**

	*U*^11^	*U*^22^	*U*^33^	*U*^12^	*U*^13^	*U*^23^
O1	0.0423 (10)	0.0304 (8)	0.0244 (7)	−0.0098 (7)	0.0009 (7)	0.0025 (7)
N1	0.0314 (9)	0.0243 (9)	0.0226 (8)	−0.0053 (8)	0.0002 (7)	0.0004 (7)
N2	0.0447 (11)	0.0279 (9)	0.0198 (8)	−0.0126 (8)	0.0022 (8)	−0.0015 (7)
C1	0.0282 (11)	0.0261 (10)	0.0184 (9)	−0.0014 (9)	−0.0009 (8)	−0.0002 (8)
C2	0.0282 (11)	0.0334 (11)	0.0187 (9)	−0.0055 (9)	0.0016 (8)	−0.0002 (8)
C3	0.0422 (13)	0.0422 (13)	0.0230 (10)	−0.0154 (11)	0.0041 (10)	−0.0059 (10)
C4	0.0440 (14)	0.0596 (17)	0.0199 (10)	−0.0188 (13)	0.0004 (10)	−0.0054 (10)

### Geometric parameters (Å, °)

**Table:**

O1—H1	0.852 (13)	C2—C2^i^	1.390 (4)
N1—C1	1.289 (3)	C2—C3	1.388 (3)
N1—O1	1.422 (2)	C3—C4	1.373 (3)
N2—C1	1.359 (2)	C3—H3A	0.9300
N2—C2	1.398 (3)	C4—C4^i^	1.380 (5)
N2—H2A	0.8600	C4—H4A	0.9300
C1—C1^i^	1.481 (4)		
			
N1—O1—H1	101.8 (17)	C3—C2—N2	121.7 (2)
C1—N1—O1	109.96 (16)	C2^i^—C2—N2	118.68 (11)
C1—N2—C2	123.50 (18)	C4—C3—C2	120.1 (2)
C1—N2—H2A	118.3	C4—C3—H3A	120.0
C2—N2—H2A	118.3	C2—C3—H3A	120.0
N1—C1—N2	125.13 (19)	C3—C4—C4^i^	120.25 (14)
N1—C1—C1^i^	117.89 (12)	C3—C4—H4A	119.9
N2—C1—C1^i^	116.98 (12)	C4^i^—C4—H4A	119.9
C3—C2—C2^i^	119.62 (13)		
			
C1—N2—C2—C3	−177.8 (2)	C2—N2—C1—C1^i^	−11.4 (4)
C1—N2—C2—C2^i^	2.2 (4)	C2^i^—C2—C3—C4	−2.1 (4)
O1—N1—C1—N2	0.8 (3)	N2—C2—C3—C4	177.9 (2)
O1—N1—C1—C1^i^	−178.7 (2)	C2—C3—C4—C4^i^	0.4 (5)
C2—N2—C1—N1	169.1 (2)		

Symmetry codes: (i) −*x*+5/4, −*y*+1/4, *z*.

### Hydrogen-bond geometry (Å, °)

**Table:**

*D*—H···*A*	*D*—H	H···*A*	*D*···*A*	*D*—H···*A*
O1—H1···N1^ii^	0.852 (13)	1.971 (10)	2.763 (2)	154
N2—H2A···O1	0.86	2.25	2.566 (3)	102

Symmetry codes: (ii) −*x*+1, −*y*, −*z*+1.
